# Insight from expression profiles of *FT* orthologs in plants: conserved photoperiodic transcriptional regulatory mechanisms

**DOI:** 10.3389/fpls.2024.1397714

**Published:** 2024-06-03

**Authors:** Nayoung Lee, Jae Sung Shim, Min-Kyoung Kang, Moonhyuk Kwon

**Affiliations:** ^1^ Research Institute of Molecular Alchemy (RIMA), Gyeongsang National University, Jinju, Republic of Korea; ^2^ School of Biological Sciences and Technology, Chonnam National University, Gwangju, Republic of Korea; ^3^ Division of Applied Life Science (BK21 Four), Anti-aging Bio Cell factory Regional Leading Research Center (ABC-RLRC), Gyeongsang National University, Jinju, Republic of Korea; ^4^ Division of Applied Life Science (BK21 Four), ABC-RLRC, RIMA, Gyeongsang National University, Jinju, Republic of Korea

**Keywords:** photoperiodic flowering, FLOWERING LOCUS T, florigen, expression profiles, flowering plants

## Abstract

Floral transition from the vegetative to the reproductive stages is precisely regulated by both environmental and endogenous signals. Among these signals, photoperiod is one of the most important environmental factors for onset of flowering. A florigen, *FLOWERING LOCUS T* (*FT*) in *Arabidopsis*, has thought to be a major hub in the photoperiod-dependent flowering time regulation. Expression levels of *FT* likely correlates with potence of flowering. Under long days (LD), *FT* is mainly synthesized in leaves, and FT protein moves to shoot apical meristem (SAM) where it functions and in turns induces flowering. Recently, it has been reported that *Arabidopsis* grown under natural LD condition flowers earlier than that grown under laboratory LD condition, in which a red (R)/far-red (FR) ratio of light sources determines *FT* expression levels. Additionally, *FT* expression profile changes in response to combinatorial effects of FR light and photoperiod. FT orthologs exist in most of plants and functions are thought to be conserved. Although molecular mechanisms underlying photoperiodic transcriptional regulation of *FT* orthologs have been studied in several plants, such as rice, however, dynamics in expression profiles of *FT* orthologs have been less spotlighted. This review aims to revisit previously reported but overlooked expression information of *FT* orthologs from various plant species and classify these genes depending on the expression profiles. Plants, in general, could be classified into three groups depending on their photoperiodic flowering responses. Thus, we discuss relationship between photoperiodic responsiveness and expression of *FT* orthologs. Additionally, we also highlight the expression profiles of *FT* orthologs depending on their activities in flowering. Comparative analyses of diverse plant species will help to gain insight into molecular mechanisms for flowering in nature, and this can be utilized in the future for crop engineering to improve yield by controlling flowering time.

## 1 Introduction

Plants have evolved their flowering strategies to maximize reproductive success. Both environmental and endogenous signals influence the timing of flowering via integration in photoperiod, temperature, vernalization, aging, phytohormone, autonomous pathways, and carbohydrate metabolism (Teotia and Tang, 2015; Rehman et al., 2023). Among these signals, photoperiod is considered as the most crucial environmental cue for flowering time regulation, as it is a stable indicator of the seasonal changes. Indeed, photoperiod-dependent flowering regulation is likely conserved across angiosperms. Flowering plants can be categorized into three major groups based on photoperiodic responses for flowering induction: short day plants (SDPs), long day plants (LDPs), and day-neutral plants (DNPs). SDPs flower when the daylength is at or shorter than a certain length, while flowering in LDPs is induced as daylength increases. Flowering in DNPs is not dependent on photoperiod. Photoperiodic flowering regulation has been extensively studied in a model plant, *Arabidopsis*, which is a representative LDP. The onset of flowering is determined by the expression levels of *FT* (Krzymuski et al., 2015). *FT* mRNA is almost undetectable in unfavored SD conditions. However, *FT* expression gradually increases as the daylength increases. *FT* is mainly synthesized in a subset of phloem companion cells in the distal part of leaves (Takada and Goto, 2003; Chen et al., 2018). *Arabidopsis* recognizes the different photoperiods and rapidly determines the expression of *FT*. When SD-grown plants are transferred to LD, *FT* expression is promoted on the first day of transfer (Krzymuski et al., 2015). In line with this, *Arabidopsis* flowering can be promoted with exposure to a single LD light regime. It should be noted that, despite the positive correlation between *FT* expression and the onset of flowering time, FT-mediated flowering is not solely dependent on *FT* expression in leaves. Since *FT* is mainly transcribed and synthesized in leaves and then FT protein moves from leaves to the shoot apical meristem (SAM), where it functions to induce flowering, the amount of FT protein translocated into SAM could be determined by several regulatory mechanisms including stability of *FT* mRNA (Seo et al., 2011; Wu et al., 2013), the success of FT transport processes (such as uploading to and unloading from the phloem) (Liu et al., 2012; Zhu et al., 2016; Endo et al., 2018; Susila et al., 2021), the stability and activity of FT protein (Qin et al., 2017; Xia et al., 2020), etc.

**Figure 1:**
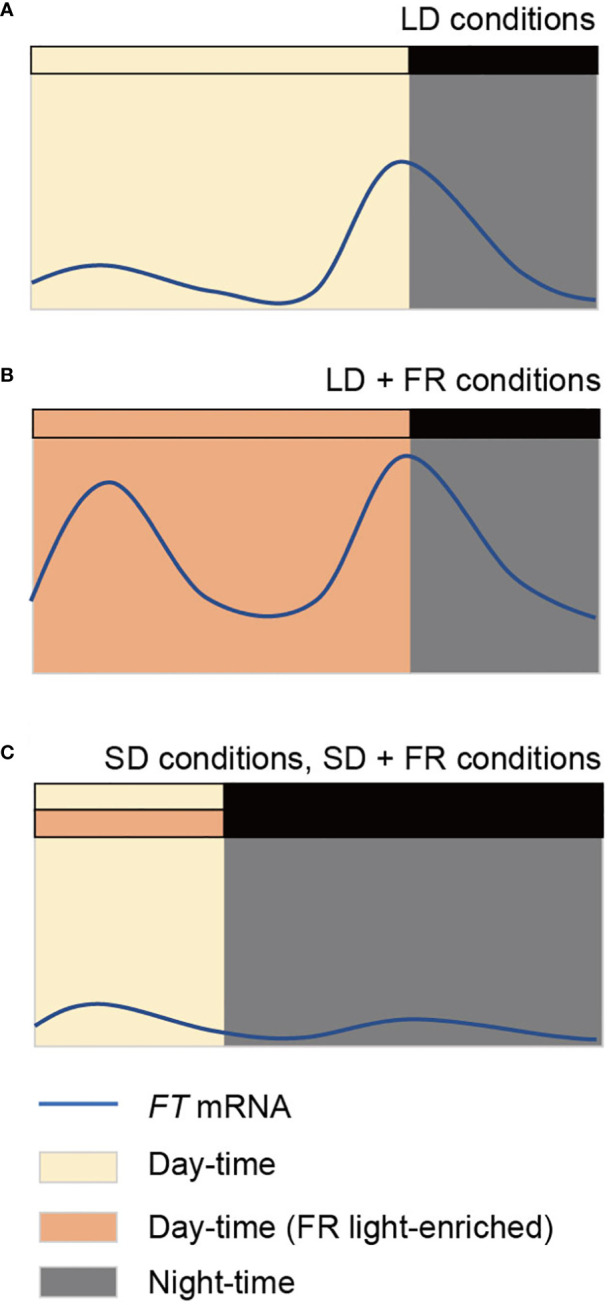
Diurnal expression profiles of *FLOWERING LOCUS T* (*FT*) in *Arabidopsis* seedlings. *FT* expression peaks once in a day at the end of day under laboratory long day (LD) conditions **(A)**. Under natural LDs or when far-red (FR) light is supplemented in LD conditions (LD + FR), expression of *FT* peaks twice in a day in the morning and the evening **(B)**. *FT* expression is low in short day (SD) conditions, both in the absence and presence of supplementary FR light (SD+FR) **(C)**. Yellow and orange boxes indicate day period without and with supplementary FR light, respectively. Night period is indicated as dark or grey boxes.

In addition to the photoperiodic regulation, *FT* expression levels are regulated in a time-of-day-dependent manner. *FT* expression peaks at the end of daytime (dusk) under laboratory LD conditions (**Figure 1A**). A B-box (BBX) transcription factor, CONSTANS (CO), is a major transcription factor activating *FT* expression under LDs. Overall expression profiles of *CO* are similarly maintained regardless of photoperiod, as *CO* expression is controlled by the circadian clock (Shim et al., 2017). In contrast, the stability of CO protein is regulated by light conditions. Combined with the circadian clock-mediated transcriptional regulation of *CO* expression and light-dependent CO protein stability, CO can accumulate only in LD conditions, consequently inducing *FT* expression and flowering. This regulatory mechanism of photoperiodic flowering fits well with the external coincidence model (Pittendrigh and Minis, 1964). Although CO is one of the most important factors in this photoperiodic regulation of *FT* expression, numerous factors have been identified as regulators of *FT* expression. A recent review has summarized the complicated regulatory networks involved in photoperiod-dependent *FT* expression (Takagi et al., 2023).

Interestingly, a recent study has demonstrated that the *FT* expression profile differs when *Arabidopsis* grows in natural LD conditions compared in laboratory LDs, without altering the vasculature-specific expression pattern (Song et al., 2018) (**Figure 1B**). The expression of *FT* peaks in the morning as well as in the evening. The ratio of R/FR light is responsible for the variation in *FT* expression profiles between natural LDs and laboratory LDs. Typical laboratory conditions with fluorescent light sources have higher R/FR ratios than those of sunlight which has a R/FR ratio of approximately 1 (Holmes and Smith, 1977; Song et al., 2018). Laboratory LD conditions with supplementary FR light, mimicking the sunlight R/FR ratio, can create the bimodal *FT* expression profile and accelerate flowering (Song et al., 2018). In contrast, laboratory SD conditions with a R/FR ratio of 1 fail to induce *FT* expression (**Figure 1C**), suggesting that the regulation of *FT* expression in nature is still photoperiod-dependent. Among the previously identified *FT*-regulating factors, a R/FR light photoreceptor, phytochrome A (phy A), is necessary for *FT* expression especially in the morning. phyA induces *FT* expression partially via the stabilization of CO protein and increasing the activity of phytochrome interacting factors (PIFs) (Song et al., 2018; Lee et al., 2023). Although the supplementary FR light (adjusted R/FR ratio signal) in LD induces *FT* expression levels both in the morning and at dusk, they are likely regulated by different molecular mechanisms. Exposure to supplementary FR light does not immediately induce *FT* expression in the morning, while it rapidly induces the evening *FT* expression, suggesting that long-term transmission of light signal is required for the morning *FT* induction (Lee et al., 2023). This may explain the photoperiodic regulation of *FT* expression under FR light-enriched conditions. Nevertheless, detailed molecular mechanisms need to be elucidated.

**Figure 2:**
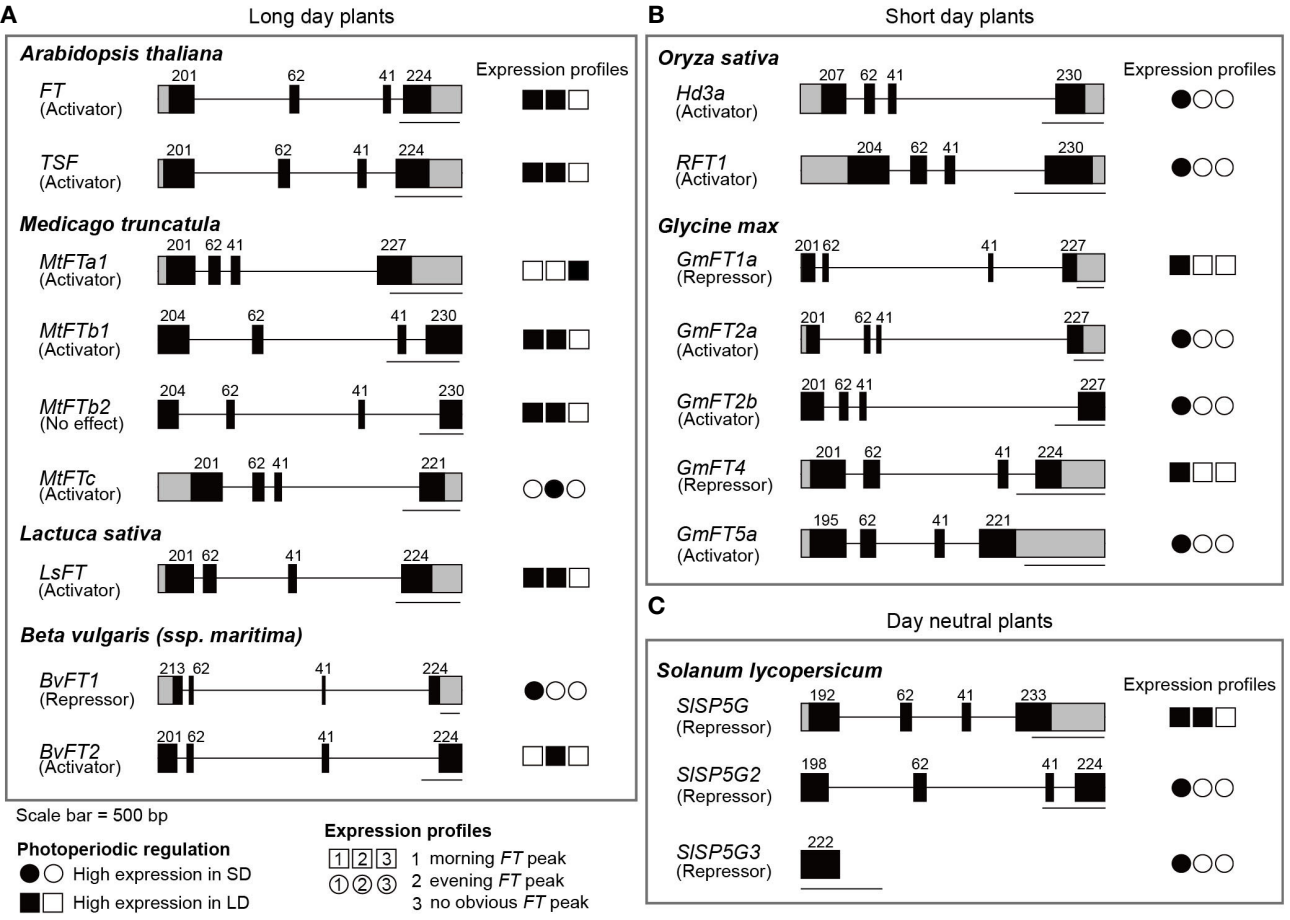
Gene structures and photoperiod-dependent expression profiles of *FT* and its ortholog genes. The gene structures of *FT* and *FT* orthologs in LDPs **(A)**, SDPs **(B)**, and a DNP **(C)** are indicated with their respective photoperiod-dependent expression profiles. Floral activities are denoted below the *FT* orthologs in parenthesis. In the gene structures, black and grey boxes represent exons and UTRs, respectively, and numbers above the exons indicate size of each exon in base pairs. A set of three circles or rectangles indicates expression patterns of *FT* ortholog in either SD or LD, depending on the photoperiod in which expression levels are high. The photoperiod-dependent expression profiles are denoted by the shape type; circles mean higher expression in SD than in LD, while rectangles mean higher expression in LD than in SD. Time-of-day expression profiles are indicated by the position of filled shapes; filled circles or rectangles denote expression peaks observed in the morning (position 1) or in the evening (position 2). A filled circle or rectangle at position 3 indicates that a gene is expressed irrespective of the time of day. Scale bar = 500 bp.

The determination of when to flower is crucial for reproductive success, impacting crop yield including grain yield and biomass (Blümel et al., 2015). Therefore, understanding the photoperiodic flowering of various plant species is essential. It has been widely accepted that *FT* orthologs are evolutionally and functionally conserved in flowering plants, and the expression of *FT* orthologs is required to promote flowering, regardless of whether a plant is a SDP, LDP, or DNP. Given the observed differences in *FT* expression profiles in *Arabidopsis* depending on light quality, especially R/FR ratios, it prompts us to investigate *FT* orthologs in diverse plant species to understand the significance of expression profiles on flowering in nature. Additionally, while *FT* orthologs in many plant species share similar gene structures in general (**Figure 2**), their functions are, in fact, diversified, playing roles either activators or repressors in floral regulation. Hence, we also focus on relationship between floral activities of FT orthologs and their expression profiles.

## 2 Conservation of expression profiles of *FT* orthologs across flowering plants

FT belongs to Phosphatidyl ethanolamine-binding protein (PEBP) family. A subfamily containing FT, named the FT-like clade, has been identified not only in angiosperms but also in gymnosperms (Jin et al., 2021). In flowering plants, the role of FT in flowering induction is evolutionally and functionally conserved. Besides its involvement in flowering time regulation, diverse roles of FT-like PEBP have been characterized, which has been summarized and reviewed (Jin et al., 2021). Although FT and its orthologs have distinct as well as shared roles, their gene structures are well-conserved (Almeida de Jesus et al., 2022; Liu et al., 2023) (**Figure 2**). Most *FT* orthologs consist of four exons, with few exceptions reported in banana and macadamia (Chaurasia et al., 2017; Yang et al., 2023). The size of exons is similar among *FT* orthologs, with the 2^nd^ and 3^rd^ exons having a consistent length (Liu et al., 2023). Conversely, the length of introns is variable, with an intron of over 3000bp present in sugar beet (*BvFT1*) and soybean (*GmFT2a*) among *FT* orthologs investigated in this review (**Figure 2**). The first intron of *Arabidopsis FT*, along with the promoter region, has been shown to be a regulatory region controlling *FT* expression in response to various environmental signals including light and temperature (Bratzel and Turck, 2015; Deng and Chua, 2015). Given that most *FT* orthologs have at least one intron longer than the average intron length of 165 bp in *Arabidopsis* (Feldmann and Goff, 2014), it is possible that mode of actions for regulation of *FT* expression by environmental changes is also conserved in various plant species.

Since a concept of florigen first arose in 1936 (Chailakhyan, 1936), extensive physiological analyses combined with molecular-genetic approaches in various plants have revealed that functionally conserved FT orthologs play key roles in flowering regulation. Regulatory mechanisms governing the expression of *FT* orthologs under diverse environmental conditions have been elucidated, particularly in model plants such as *Arabidopsis* and rice. In other plants, however, detailed regulatory mechanisms remain unclear. In this section, we compare expression profiles of *FT* orthologs in three plant groups, each exhibiting distinct responses to daylength for flowering: SDPs, LDPs, and DNPs. Additionally, we provide a summary of the effects of FR light on flowering regulation, accompanied by the expression of *FT*.

### 2.1 *FT* orthologs in LDPs

#### 2.1.1 Light quality shapes *FT* expression profiles of *Arabidopsis*

The photoperiodic regulation of *FT* in LDPs has been extensively investigated by using *Arabidopsis*. The external coincidence model well fits into the photoperiodic regulation of flowering time in *Arabidopsis*, wherein flowering is induced only when a clock-controlled internal rhythm coincides with an external factor. The expression of *CO* follows a rhythmic pattern controlled by the circadian clock, gradually increasing from the middle of the day in LD conditions (Imaizumi, 2010). This increase is facilitated by the action of GIGANTEA (GI) and FLAVIN-BINDING, KELCH REPEAT, F-BOX (FKF1). *GI* expression, also regulated by the circadian clock, peaks in the middle of the day (Fowler et al., 1999). In LD conditions, the blue light receptor FKF1 accumulates and activates in the midday, forming a complex with abundant GI protein, subsequently activating *CO* expression. The stability of CO protein is light-regulated. HIGH EXPRESSION OF OSMOTICALLY RESPONSIVE GENE1 (HOS1) and CONSTITUTIVE PHOTOMORPHOGENIC 1 (COP1)-SUPRESSOR OF PHYA-105 1 (SPA1)/-SPA3-SPA4 complex destabilize CO protein in the morning and in the night, respectively (Shim et al., 2017). In LD conditions, high *CO* expression coincides with light period, which in turn induces *FT* expression. In contrast, in SD conditions, when *CO* expression level is high, the dark period prevents CO protein stabilization and subsequent *FT* activation. This mechanistic model is based on the observed *FT* expression profile in laboratory LD/SD conditions. However, in nature, a morning *FT* peak as well as the evening *FT* peak appears. It has been shown that different R/FR ratios between sunlight (R/FR = 1) and laboratory conditions (R/FR > 2) determines the expression profiles (Song et al., 2018). Indeed, supplementary FR light in laboratory LD conditions mimics the bimodal *FT* expression profile observed in nature. Although the supplementary FR light induces the *FT* expression levels both in the morning and at dusk, the mechanisms underlying FR light-mediated *FT* induction likely differ between morning and evening. Firstly, the evening *FT* expression is induced in response to the supplementary FR light exposure as short as 2 hours (Lee et al., 2023). However, unlike induction of *PIL1* expression, a marker of shade responses enriched by FR light, the evening *FT* expression could be induced when the 2 hr supplementary FR light was irradiated 6 hr ahead of dusk. This suggests that the evening *FT* expression is influenced by light quality (R/FR ratio) and the circadian clock, which is consistent with the classical external coincidence model. Secondly, the morning *FT* induction requires prolonged FR light exposure. Neither a 2hr, 4hr, or 8hr FR light exposure is sufficient for full morning *FT* expression (Lee et al., 2023). phyA, but not phyB, has thought to be crucial for the morning *FT* induction under natural LD or FR light-enriched LD conditions. Mutation on *phyA* abolishes the morning *FT* induction under FR light supplemented LD conditions, while mutation on *phyB* does not affect the morning *FT* expression level in laboratory LD conditions (Song et al., 2018). Furthermore, end-of-day FR light treatment accelerates flowering, mediated by phyB but not phyA (Cerdán and Chory, 2003). These findings, combined with the requirement of prolonged FR light exposure, support the notion that the morning *FT* expression is a FR high-irradiance response (HIR) via phyA. As part of phyA-mediated *FT* expression regulation, PIF7, together with other PIFs including PIF4 and PIF5, directly activates *FT* expression (Lee et al., 2023). EARLY FLOWEIRING 3 (ELF3) has shown to antagonize phyA in the regulation of morning *FT* expression. *elf3* mutant significantly increased *FT* expression in the morning under both FR light-enriched LD conditions and laboratory LD conditions. ELF3 affects *FT* expression by regulating the stability of CO protein, in which ELF3 interacts with the COP1/SPA1 complex (Laubinger et al., 2006; Jang et al., 2008; Yu et al., 2008; Huang et al., 2016). Under simulated natural LD conditions, the abundance of ELF3 protein is lower compared to laboratory LD conditions, and the stability of CO protein increases throughout the day when *ELF3* is mutated (Song et al., 2018). This suggests that supplementary FR light reduces ELF3 protein stability, thereby stabilizing CO protein and resulting in morning *FT* expression. Based on previously reported *FT* regulators and their molecular mechanisms, phyA and ELF3 have been identified as regulators of morning *FT* expression. However, considering observations that none of mutants except *phyA* and *fhy1fhl* examined by Song et al., 2018 specifically affected morning *FT* expression, it is possible that the regulation of morning *FT* expression is complex. Indeed, in addition to the prolonged FR light exposure, the induction of morning *FT* expression appears to be controlled by the circadian clock, as indicated by the presence of time windows most effective for *FT* induction by the supplementary FR light (Lee et al., 2023). FR light treatment from the late afternoon in the previous day to the early morning is sufficient to fully induce morning *FT* expression. Thus, the regulation of morning *FT* expression is tightly governed by both the circadian clock and prolonged FR light signals. Further investigations are required to elucidate the molecular mechanisms underlying *FT* expression under FR light enriched LD conditions.

*Arabidopsis* plants under canopy exhibit earlier flowering compared to those fully exposed to light. They perceive shaded conditions by sensing R/FR ratios, which is mediated by photoreceptors including phys. In shade, neighboring plants absorb R light but reflect FR light, leading to a decreased amount of R and an increased FR light. As a result, the R/FR ratio varies depending on the degree of shade. In response to the reduced R/FR ratio under shade, active phys turns into inactive state, triggering shade avoidance responses including acceleration of flowering (Casal, 2012). The early flowering phenotype in shade is predominantly dependent on phyB with contributions from phyD and phyE (Devlin et al., 1999, 1998). On the other hand, phyA has thought to be less effective in flowering regulation under shade, even though it mediates other shade avoidance responses (Franklin et al., 2003; Roig-Villanova and Martínez-García, 2016). It has been shown that both morning and evening *FT* expression gradually increase as the R/FR ratio decreases from normal laboratory white light (R/FR ratio = 2.0) to shade conditions (R/FR = 0.25) (Song et al., 2018). Given that the induction of *FT* expression requires functional phyA, these observations suggest that the phyA-mediated regulatory mechanism for *FT* induction under FR-enriched LD conditions may also partially contribute to early flowering under shade conditions.

#### 2.1.2 Expression profiles of *FT* orthologs in other LDPs

*Medicago truncatula* (*Medicago*), belonging to the rosidae subclass along with *Arabidopsis*, is a model plant of legumes that can mediate nitrogen fixation. Its flowering is promoted by long days (Clarkson and Russell, 1975). *Medicago* possesses five *FT* ortholog genes, *MtFTa1*, *MtFTa2*, *MtFTb1*, *MtFTb2*, and *MtFTc*. Among these, *MtFTa1*, *MtFTb1*, and *MtFTc* exhibit flowering activation activity, as demonstrated by the complementation of the late flowering phenotype in *Arabidopsis ft* mutants upon overexpression of each *FT* ortholog gene (Laurie et al., 2011). The role of *MtFTa1* in floral induction has been further confirmed in *Medicago*, however, *mtftc* mutants did not affect flowering in *Medicago* (Laurie et al., 2011). Except for *MtFTa2*, the expression of the four *MtFT* genes is dependent on photoperiod (**Table 1**). In LD conditions, *MtFTa1*, *MtFTb1*, and *MtFTb2* are expressed, while their expression is either absent or marginal in SD conditions (Laurie et al., 2011). This implies that *MtFTa1* and *MtFTb1* are responsible for photoperiodic flowering regulation, while *MtFTb2* may contribute to photoperiodic responses other than flowering. *MtFTb1* and *MtFTb2* show bimodal expression profiles in LD conditions with peaks in the morning (Zeitgeber Time, ZT4) and at dusk (ZT16), reminiscent of the *Arabidopsis FT* expression profile observed in FR-enriched LD conditions. In contrast, *MtFTa1* expresses consistently throughout the day in LD. The expression of *MtFTc* also appears to be regulated by photoperiod, but unlike *MtFTa1*, *MtFTb1*, and *MtFTb2*, its expression level is higher in SD than in LD conditions (Laurie et al., 2011)(**Table 1**). In SD conditions, *MtFTc* expression levels start to increase in the morning and peak after dusk (during the night-time period), which is a unique pattern among the *FT* orthologs examined in this review (Laurie et al., 2011) (**Table 1**). Moreover, *MtFTc* expresses at a low level in leaves in LD conditions. This expression profile suggests that *MtFTc* may have little or weak contribution to flowering time regulation in LD, and its high expression in SD conditions implies a potential role in regulating flowering time in SD. Conversely, *MtFTa2* is expressed in both LD and SD conditions. Its expression level in LD remains relatively constant throughout the day, while it peaks after dusk in SD conditions, similar to *MtFTc*. The inability of ectopic expression of *MtFTa2* in *Arabidopsis* to rescue the late flowering of the *ft* mutant, combined with its high expression in tissues other than leaves such as roots, suggests diversified role(s) for *MtFTa2* beyond flowering time regulation in *Medicago*. In addition to *FT* orthologs, orthologs in *Medicago* of photoperiodic flowering time-regulating factors such as *CO* and *CO*-like (*COL*), *GI*, and *FKF1* genes have been identified, though their functions in flowering remain to be resolved or appear to be diversified (Hecht et al., 2005; Wong et al., 2014). *Medicago* possesses a single copy of *PHYA* gene. *MtPHYA* positively regulates flowering in LD, while its effect becomes weaker in SD, suggesting a photoperiod-dependent contribution of phyA to flowering (Jaudal et al., 2020). Despite the functional conservation of phyA between *Medicago* and *Arabidopsis* in flowering, it has been shown that flowering in *Medicago sativa* (*M. Sativa*), a close relative *Medicago truncatula*, is delayed under shade conditions. This shade-induced delay of flowering was also observed under a R/FR ratio of 0.8 (Lorenzo et al., 2019). Given the sunlight R/FR ratio close to 1, *M. Sativa* in its natural habitat may flower later than in laboratory LD conditions. Under shade with a R/FR ratio of 0.4–0.6, the expression levels of *MsFTa1* and *MsFTb1* were reduced around dusk compared to normal LD conditions, which is consistent with the reduction of expression of *SQUAMOSA PROMOTER BINDING LIKE 3* (*SPL3*), a floral activator (Lorenzo et al., 2019). However, it should be noted that floral activators such as *PIF3* and *HB2* are up-regulated in the same condition. Further analysis is needed to understand how the regulatory pathway of flowering responds to supplementary FR light. The delay in flowering under shade in *M. Sativa* is likely a diverged strategy to increase survival rates by choosing the accumulation of resources instead of flowering under unfavorable conditions, as a characteristic of a perennial plant. However, *Medicago truncatula*, an annual plant, also exhibits similar responses to shade or supplementary FR light; flowering is delayed in shaded conditions (Mauro et al., 2014), and *HB2* expression is up-regulated by supplementary FR light (Zhang et al., 2020). The delay in flowering due to shade has also been reported in another legume plant, soybean. Flowering of soybean is delayed under low R/FR light quality (Cober and Voldeng, 2001) (Discussed in Section 2.2.2). Thus, the determination of flowering time in response to different R/FR ratios may be governed by legume-specific mechanisms rather than whether it is an annual or a perennial plant.

**Table 1:** *FT* orthologs and their expression profiles in flowering plants.

Photoperiodic responses	Organism	Leaf structure	*FT* orthologs	Effect on flowering	Expression profiles^a^		Effect of phyA or shade (low R/FR) on flowering		Reference
					SD	LD	phyA	Shade or low R/FR	
LDPs	*Arabidopsis thaliana*	eudicots	*FT*	Activator	○○○	□■□ (in high R/FR) ■■□ (in low R/FR)	Activator	promote	Casal, 2012; Krzymuski et al., 2015; Lee et al., 2023; Mockler et al., 2003; Song et al., 2018
			*TSF*	Activator	○○○	□■□ (in high R/FR) ■■□ (in low R/FR)			
	*Medicago truncatula*	eudicots	*MtFTa1*	Activator	○○○	□□■	Activator	delay	Laurie et al., 2011; Mauro et al., 2014; Jaudal et al., 2020
			*MtFTa2*	No effect	○●○	□■□			
			*MtFTb1*	Activator	○○○	■■□			
			*MtFTb2*	No effect	○○○	■■□			
			*MtFTc*	Activator	○●○ (ZT12)	□□□			
	*Lactuca sativa*	eudicots	*LsFT*	Activator	n.d.	■■□	n.d.	promote	Sgamma et al., 2016; Fukuda et al., 2017; Alrajhi et al., 2023
	*Beta vulgaris*	eudicots	*BvFT1*	Repressor	●○○	□□□	n.d.	promote	Lane et al., 1965; Pin et al., 2010
			*BvFT2*	Activator	○○○	□■□			
SDPs	*Oryza sativa*	monocots	*Hd3a*	Activator	●○○	■□□ low expression	Activator (mild)	promote	Ikeda, 1985; Izawa et al., 2002; Kojima et al., 2002; Tamaki et al., 2007; Komiya et al., 2008
			*RFT1*	Activator	●○○	■□□ low expression			
	*Glycine max*	eudicots	*GmFT1a*	Repressor	○○○	■□□	Repressor	delay	Cober et al., 1996; Cober and Voldeng, 2001; Lee et al., 2021
			*GmFT1b*	Repressor	n.d.	n.d.			
			*GmFT2a*	Activator	●○○	□□□			
			*GmFT2b*	Activator	●○○	□□□			
			*GmFT3a*	Activator	n.d.	n.d.			
			*GmFT3b*	Activator	n.d.	n.d.			
			*GmFT4*	Repressor	○○○	■□□			
			*GmFT5a*	Activator	●○○	□□□			
			*GmFT5b*	Activator	n.d.	n.d.			
			*GmFT6*	Repressor	●○○	□□□			
DNPs	*Solanum lycopersicum*	eudicots	*SFT* (*SP3D*)	Activator	○○●	■□□	n.d.	both	Cao et al., 2018, 2016; Kalaitzoglou et al., 2019; Meijer et al., 2023
			*SP5G*	Repressor	○○○	■■□			
			*SP6A*	n.d.	Not expressed				
			*SP5G1*	n.d.	Not expressed				
			*SP5G2*	Repressor	●○○	■■□ low expression			
			*SP5G3*	Repressor	●○○	□□■ low expression			

a expression profiles in SD and LD conditions are denoted by a set of three circles (SD) or rectangles (LD). Filled circles or rectangles indicate expression peaks observed in the morning (first) or in the afternoon (second), and the third circle or rectangle indicates that a gene is expressed time-of-day independently. low expression means overall expression levels are lower in LD compared to those in SD.

n.d., not determined.

*Lactuca Sativa* (Lettuce), a dicot like *Arabidopsis*, belongs to the asteridae subclass, and its flowering is induced with longer day lengths. Lettuce has been shown to have one copy of the *FT* ortholog gene, *LsFT*, which functions as a floral activator (Fukuda et al., 2011) (**Table 1**). While it remains unclear whether photoperiod affects *LsFT* expression levels, the rates of its accumulation during development in different cultivars positively correlate with floral induction (Fukuda et al., 2017), further supporting the functional conservation of FT ortholog in Lettuce. Under LD conditions, *LsFT* exhibits high expression in the morning and around dusk (Sgamma et al., 2016), resembling the expression pattern observed in *Arabidopsis* grown under FR light-enriched LD conditions. However, this result was obtained from lettuce grown in a greenhouse, thus it needs to be elucidated if the expression profile of *LsFT* is also controlled by FR light that is enriched in natural light conditions. Nevertheless, the sole *FT*-like gene in lettuce, *LsFT*, behaves similarly to *Arabidopsis FT*. FR light likely accelerates flowering in lettuce, as supplementary FR light in the presence of R/Blue/Green light increased stalk heights, indicative of a premature flowering response (Alrajhi et al., 2023). However, little is known whether *LsFT* is responsible for floral induction by the supplementary FR light. Being one of the most popular leafy vegetables, delayed flowering in lettuce is preferred to prevent stem elongation and bitterness (Assefa et al., 2019). Therefore, understanding the flowering time pathway of lettuce is crucial.

*Beta vulgaris* (Beet) belongs to Caryophyllidae, the third-largest subclass of the class Magnoliopsida (dicotyledons). Beet possesses two *FT*-like genes, *BvFT1* and *BvFT2*, which interestingly exhibit opposing functions in flowering regulation. *BvFT2* promotes flowering, while *BvFT1* represses flowering in both beet and *Arabidopsis* when ectopically expressed (Pin et al., 2010) (**Table 1**). The antagonistic roles of *BvFT*s appear to be conferred by sequence variations in segment B of the fourth exon, encoding an external loop of PEBP proteins. In this segment, both BvFT proteins share a conserved key amino acid, Glutamine (Q), at residue 144 and 140 in BvFT1 and BvFT2, respectively, that differentiates FT from its close PEBP protein TFL1, a floral repressor (Ahn et al., 2006). However, only BvFT2 has sequence conservation throughout the entire segment B. Indeed, swapping the segment B sequences in BvFTs altered their inherent activity in opposite ways (Pin et al., 2010). In addition to their opposing roles, expression profiles of the two *BvFTs* differ. Similar to *Arabidopsis FT* in laboratory conditions, *BvFT2* expresses high in LD but not in SD conditions with a peak around dusk in annual beets (Pin et al., 2010). On the contrary, *BvFT1* is expressed highly in SD with a peak after dawn (Pin et al., 2010). In biennial beets without vernalization, the expression of *BvFTs* is photoperiod-independently regulated. *BvFT1* expresses in both SD and LD with the same diurnal profile, while *BvFT2* does not express, which is consistent with essential role of vernalization for floral induction in biennial plants. After vernalization, the expression profiles of each *BvFT*s resemble those in annual beets. As a root crop, delaying flowering in the first year of the biennial plant’s life cycle is necessary for increasing leaf and root mass for sugar and bioethanol production. Several flowering-regulating genes have been identified and characterized in the regulation of beet flowering, such as *BOLTING TIME CONTROL 1* (*BvBTC1*), a key determinant of vernalization requirements between annual and biennial plants (Pin et al., 2012). As a positive regulator of flowering, BvBTC1 is likely involved in the responsiveness to LD, as shown that reduced *BvBTC1* expression by RNAi caused induction of *BvFT1* expression and reduction of *BvFT2* expression (Pin et al., 2012). Additionally, several reports have demonstrated the negative effect of shade on leaf and root biomass of beet (Artru et al., 2018; Schambow et al., 2019). In 1965, Lane et al. showed that by using different types of lamps, flowering time varied depending on R/FR ratios. In general, supplementary FR light promotes flowering in beets in the presence of R light. Thus, beet determines flowering time in response to the light quality, likely via mechanisms similar to phyA-mediated flowering regulation in *Arabidopsis*.

### 2.2 *FT* orthologs in SDPs

#### 2.2.1 Two *FT* orthologs, *Hd3a* and *RFT1*, in rice

The mechanisms governing the photoperiodic regulation of flowering in SDPs have been demonstrated mostly in rice, one of the world’s major crops. Rice, a monocotyledonous flowering plant in the grass family Poaceae, initiates flowering in response to daylengths shorter than 13.5h (Itoh et al., 2010). In rice, two *FT*-like genes, *Heading date 3a* (*Hd3a*) and *RICE FT1* (*RFT1*), play crucial roles as mobile florigens synthesized in leaves and essential for flowering (Kojima et al., 2002; Tamaki et al., 2007; Komiya et al., 2008) (**Table 1**). The expression of these florigens is responsive to the photoperiod, with higher expression levels observed in SD conditions. Under SD conditions (10 h light, 30°C/14 h dark, 25°C), the expression of *Hd3a* and *RFT1* peaks at or before dawn, and then gradually decrease during the day (Izawa et al., 2002; Komiya et al., 2008). The diurnal expression profiles of *Hd3a* and *RFT1* are regulated by the activity of OsGI, whose expression is under the control of the circadian clock with a peak at the end of day (Hayama et al., 2003, 2002; Lee and An, 2015). OsGI is necessary for the diurnal expression of *HEADING DATE 1* (*Hd1*), a rice ortholog of *CO*, which activates the expression of *Hd3a* and *RFT1* in the morning (Hayama et al., 2003). This pathway resembles the CO-FT pathway in *Arabidopsis*. Additionally, a rice-specific pathway has been identified. *EARLY HEADING DATE 1* (*Ehd1*) encoding a B-type response regulator is upregulated by OsGI when blue light coincides with the morning phase, and activates *Hd3a* and *RFT1* expression (Doi et al., 2004; Itoh et al., 2010). *Ehd1*-mediated flowering regulation pathway has thought to be absent in *Arabidopsis*, although its homologs are found in *Arabidopsis* and other plants (Bar et al., 2008). It plays a central role in photoperiod-mediated flowering in rice, which is recently extensively reviewed in Vicentini et al., 2023. Conversely, in LD conditions, *Ehd1* expression is repressed by *GRAIN NUMBER, PLANT HEIGHT AND HEADING DATE 7* (*Ghd7*), encoding a CCT-domain protein highly expressed in LD (Xue et al., 2008; Itoh et al., 2010). Despite lower expression levels of *Hd3a* and *RFT1* in LD conditions compared to SD conditions, *RFT1* has shown to be a major floral activator under LD conditions (Komiya et al., 2009). Indeed, *RFT1* shows diurnal expression profile with a peak in the early morning and gradually increasing over the night (Komiya et al., 2009). The current model of photoperiodic flowering regulation mediated by *Hd3a* and *RFT1* relies primarily on their on-off gene expression profiles between SD and LD, respectively. As the photoperiod lengthens in LD conditions, high expression of *Hd1* coincides with the daytime phase. However, in contrast to the role of CO in *Arabidopsis*, Hd1 acts as a repressor for the expression of *Hd3a* and *RFT1* in LD conditions (Hayama et al., 2003). Consequently, the accumulation of Hd1 suppresses the expression of *Hd3a* and *RFT1*, leading to reduced expression around dusk. Nevertheless, this model cannot explain the circadian clock-regulated *RFT1* expression in LD, which is important for flowering in LD. The similar diurnal expression profiles of *RFT1* between SD and LD suggest that photoperiodic regulation in rice differs from that in *Arabidopsis*. *Ehd1* expression in LD showed similar expression patterns with *RFT1* (Komiya et al., 2009), thus release of suppression of *Ehd1* expression in LD conditions may accomplish with developmental processes.

FR light has been identified as a positive regulator flowering in rice, as demonstrated by experiments such as 2-hr FR light irradiation before a 10-hr dark period in LD conditions (14-hr light/10-hr dark) promoting flowering (Ikeda, 1985). In *Arabidopsis*, end-of-day (EOD)-FR light treatment counteracts the negative impact of R light by inactivating phyB (Smith, 1982; Franklin and Whitelam, 2005). Although the induction of flowering in *Arabidopsis* by EOD-FR light treatment is not solely dependent on phyB (Bagnall et al., 1995; Xie et al., 2020), it is possible that the 2-hr FR light irradiation before the dark period affects phyB activity in rice for floral induction. This hypothesis is supported by evidence that EOD-FR in SD conditions (10-hr light/14-hr dark) accelerated flowering in rice in a phyB-dependent manner (Takano et al., 2005). Another possibility is that the FR light signal itself, perceived by photoreceptors like phyA, plays a role in mediating flowering promotion, similar to *Arabidopsis*. Several observations support this idea: 1) 3-hr FR light was more effective at inducing flowering than 1-hr or 2-hr light treatment before the dark period in LD, and this FR light irradiation was more effective than extending the dark-period (Ikeda, 1985), 2) *phyA* mutant consistently exhibited delayed flowering in SD conditions (10-hr light/14-hr dark) (Takano et al., 2005; Ishikawa et al., 2009), which is different from the action of phyB, 3) simulated shade conditions accelerated flowering time, inducing *RFT1* but not *Hd3a* expression (Cui et al., 2023). Given that phyA’s function in FR high-irradiance response (HIR) has been reported in photomorphogenesis in rice (Takano et al., 2005), similarly to *Arabidopsis*, these findings suggest a role of phyA in responding to prolonged FR light in flowering time regulation through the modulation of *RFT1* (and *Hd3a*) expression.

#### 2.2.2 Expression profiles of *FT* orthologs in other SDPs

Soybean (*Glycine max*) belongs to the rosidae subclass together with *Arabidopsis* and *Medicago*, however, unlike *Arabidopsis* and *Medicago*, its flowering is induced under short days. Soybean possesses ten *FT*-like genes. *GmFT2a*/*2b*, *GmFT3a*/*3b*, *GmFT5a*/*5b* are known to activate flowering, while *GmFT1a*/*1b*, *GmFT4*, and *GmFT6* function as floral repressors (Lee et al., 2021) (**Table 1**). Their expression is controlled by photoperiod (Lee et al., 2021). The floral activators, *GmFT2a*/*2b* and *GmFT5a*, exhibit high expression levels in SD, peaking in the morning. On the other hand, *GmFT1a* and *GmFT4*, the floral repressors, are suppressed in SD but highly induced in LD. Interestingly, despite *GmFT1a* and *GmFT4* showing high expression in LD conditions, their expression peaks are observed only in the morning. This is in contrast to *FT* orthologs of LDPs, where expression peaks either once dusk (e.g., *Arabidopsis FT* in the laboratory LD conditions) or twice in the morning as well as in the dusk (e.g., *Arabidopsis FT* in natural LD conditions). Moreover, in contrast to the role of phyA in flowering in *Arabidopsis*, soybean phyAs (GmPHYA2 and GmPHYA3) are known to negatively regulate flowering time. They achieve this by increasing the expression of the legume-specific regulator *E1*, which, in turn, represses the expression of the floral activators *GmFT2a* and *GmFT5a* while activating the expression of the floral repressors *GmFT1a* and *GmFT4* (Zhai et al., 2014; Cao et al., 2017; Liu et al., 2018). *E1* is specifically expressed in LD, but not in SD, with two peaks at both dawn and dusk in LD conditions (Xia et al., 2012), resembling the expression pattern of *FT* in *Arabidopsis* plants grown under FR light enriched-LD conditions. GmPHYAs promote *E1* expression, partially through the regulation of circadian clock genes, the *GmPRR3a*/*GmPRR3b*(*Tof11*/*Tof12*)-*GmLHY* module. GmPHYs upregulate the expression of *GmPRR3a*/*GmPRR3b*, which then directly represses the expression of *GmLHY* genes. Subsequently, this relieves GmLHY-mediated suppression of *E1*, inducing its expression (Lu et al., 2020). Notably, mutations in *GmLHY* genes (*lhy1a lhy1b lhy2a lhy2b*) induces *E1* expression at dusk in LD, while the morning *E1* expression remains unaffected in the mutant (Lu et al., 2020), implying that *E1* expression in the morning and at dusk may be regulated by at least two different modes of action, similar to the regulation of *Arabidopsis FT* expression. The soybean case study suggests that although the activity of *FT* orthologs has diversified as either activators or repressors, regulatory mechanisms for bimodal expression in flowering may have been conserved through the combined action of photoperiod and circadian clock. In addition to the negative effect of phyA on soybean flowering, low R/FR ratios differentially affect soybean flowering compared to *Arabidopsis* (**Table 1**). Although shade avoidance responses such as elongation of seedlings and stem, and reduction of leaf area and size are observed in soybeans under various growth conditions similar to those in *Arabidopsis* (Green-Tracewicz et al., 2012; Yang et al., 2014; Wu et al., 2017), onset of soybean flowering in LD (20 h Light/4 h Dark) is delayed as the R/FR ratio decreases, in which *E7* is required (Cober and Voldeng, 2001). Under LD (20 h Light/4 h Dark) with a R/FR ratio of 2.7, flowering time in near-isogenic lines with *E7* allele and *e7* allele is indistinguishable. However, when the R/FR ratio is similar to or lower than the sunlight R/FR ratio, soybean lines with the *E7* allele significantly delay flowering compared to those with the *e7* allele. Although a low R/FR ratio in SD (12 h Light/12 h Dark) slightly delays flowering, the responsiveness is much weaker compared to that in LD. As phyA is thought to mainly function in LD to repress flowering in soybean, the different responsiveness to R/FR ratios between LD and SD may be due to activity of phyA. Not only soybeans but also other legume plants, including winged bean (*Psophocarpus tetragonolobus*) (Raai et al., 2020) and *Medicago* (as described in Section 2.1.2), have shown to delay flowering in response to shade. Lentil (*Lens culinaris*) have been reported to flower earlier in low R/FR conditions than in high R/FR conditions (Yuan et al., 2017), however, it should be noted that the R/FR ratio in this low R/FR condition is 1.6, even higher than the sunlight R/FR ratio. Thus, it is possible that legumes can induce a series of typical shade avoidance responses but not early flowering. Instead, they may have evolved to delay flowering under shade in a legume-specific manner.

### 2.3 *FT* orthologs in tomato, a day neutral plant

*Solanum lycopersicum* (tomato) belongs to the Asteridae subclass, along with lettuce. Tomato is typically classified as a DNP (Mizoguchi et al., 2007). Six *FT*-like genes in tomato have been identified (**Table 1**). Among them, *SINGLE-FLOWER TRUSS/SELF-PRUNING 3D* (*SFT*/*SP3D*) functions to induce flowering, while *SP5G*, *SP5G2*, *SP5G3* function as floral repressors (Cao et al., 2018, 2016). *SFT* expresses photoperiod-independently, which is consistent of tomato being a DNP. However, its expression profiles differ between SD and LD conditions. Expression levels of *SFT* are relatively stable in SD, while it peaks in the morning in LD (Cao et al., 2016). Unlike *SFT*, the expression of three floral repressors, *SP5G*, *SP5G2*, and *SP5G3*, is photoperiod-dependently regulated. Expression of *SP5G* is highly expressed in LD, but not in SD, with two peaks in the morning and the evening (Cao et al., 2016), similar to the expression profiles of *Arabidopsis FT* and *FT* orthologs acting as floral activators in LDPs. Conversely, *SP5G2* and *SP5G3* are highly expressed in the morning in SD conditions. The diurnal expression profiles of *SP5G2* and *SP5G3* resemble those of the *FT*-like genes in SDPs and the floral repressor of beet, *BvFT1* (**Figure 2**, **Table 1**). Although tomato flowering is regulated photoperiod-independently, the diurnal expression profiles of *SP5G*, *SP5G2*, and *SP5G3* imply that photoperiodic regulation of gene expression exists in tomato.

Compared to the significant contribution of phytochromes and other photoreceptors in *Arabidopsis* to flowering regulation (Takagi et al., 2023), the effects of tomato photoreceptors on flowering time have been considered much weaker, with no obvious flowering phenotype observed in mutants of the photoreceptors, except for the blue light photoreceptors, *cryptochrome 1a* (*cry1a*)/*cry2* mutant (Fantini et al., 2019). Indeed, the effect of supplementary FR light on tomato flowering varies depending on experimental conditions. Under a 12 h Light/12 h Dark cycle photoperiod, tomatoes flower later in low R/FR (0.6) ratio conditions than in conditions with R/FR ratios of 7.4 or 1.2 (Cao et al., 2018). In line with this, the expression of *FT*-like floral repressors, *SP5G* and *SP5G2*, is up-regulated. However, the delay in tomato flowering in this condition is mainly dependent on phyB1 rather than phyA. On the other hand, tomatoes grown in LD (16 h Light/8 h Dark) conditions with supplementary FR light in addition to broad-spectrum lighting in a greenhouse flower earlier than those without FR light (Meijer et al., 2023). Similarly, under R light-abundant LD conditions (16 h Light/8 h Dark, a mixture of 95% R light and 5% B light), the onset of flowering is accelerated in response to an increase in supplementary FR light (Kalaitzoglou et al., 2019). Given that the small change in R/FR ratios from the laboratory (>2.5) to sunlight (c. 1) results in different *FT* expression profiles in *Arabidopsis*, the different response of flowering may be attributed to the different experimental conditions with or without exposure to sunlight.

## 3 Future perspectives

The extensive study of FT orthologs has primarily focused on their functional conservation in flowering time regulation. However, the significance of their expression profiles has received less attention. The investigation of *FT* orthologs across various plants indicates that there may be conserved regulatory mechanisms underlying their expression. To advance our understanding in this area, future research should aim to establish rules governing the expression profiles of *FT* orthologs. This could involve:

### 3.1 Balance between activators and repressors in *FT* orthologs

FT-like PEBP proteins behave either as floral activators or repressors, with a conserved amino acid distinguishing FT orthologs from the floral repressor TFL1. Apart from sequence disparities, their expression profiles and regulatory mechanisms differ significantly. *FT* is expressed in leaves, and FT protein moves to the SAM. Conversely, *TFL1* is expressed in the center of the SAM to repress flowering (Conti and Bradley, 2007; Baumann et al., 2015). In addition to spatial regulation, *FT* expression is temporally controlled in response to photoperiod as described above, while little is known about photoperiod dependency of *TFL1* expression (Higuchi et al., 2013; Wickland and Hanzawa, 2015). The expression of *FT*-like floral repressors, such as *BvFT1* in beet, *Gm1a* and *GmFT4* in soybean, and *SP5G*, *SP5G2*, and *SP5G3* in tomato, is photoperiod-dependent. Under non-facultative photoperiod conditions, the expression of these floral repressors, except for *GmFT6* in soybean, tends to increase. This tendency is consistent with their function to repress flowering. Conversely, the expression of *FT*-like floral activators, except for *MtFTc* in *Medicago*, increased only under facultative photoperiod conditions. This implies that expression of both *FT*-like floral activators and repressors is under photoperiodic control, contributing the balance to determine flowering. The FT-like floral repressor in tobacco, NtFT2, interacts with *Arabidopsis* FD (Harig et al., 2012), suggesting that FT-like floral repressors, in addition to the floral activators, likely bind to FD orthologs. The floral repressor TFL1, which competes with FT in the SAM to repress flowering in *Arabidopsis*, also binds to FD protein (Abe et al., 2005; Wigge et al., 2005; Zhu et al., 2020). However, it is noted that the expression of *TFL1* is restricted to the SAM. The *FT*-like floral repressors are thought to be expressed in leaves like the *FT*-like floral activators. In soybean, *GmFT1a* and *GmFT4* are specifically expressed in cotyledons and trifoliate and unifoliate leaves (Liu et al., 2018; Lee et al., 2021). *BvFT1* is also mainly expressed in leaves (Pin et al., 2010), and *SP5G*, *SP5G2* and *SP5G3* in tomato are expressed high in leaves and cotyledons (Cao et al., 2016). However, translocation of these *FT*-like floral repressors from leaves to the SAM through transporters similar to those responsible for FT-like floral activators, such as FT-INTERACTING PROTEIN 1 (FTIP1) and SODIUM POTASSIUM ROOT DEFECTIVE 1 (NaKR1) in *Arabidopsis* (Liu et al., 2012; Zhu et al., 2016), remains unexplored. Considering the similarity in expression profiles among the *FT* orthologs in the same plant, competition between activators and repressors may begin in the cells where they express. Thus, comprehensive investigations into the transcriptional regulatory mechanisms of multiple FT-like floral regulators are essential for understanding flowering in various plants.

### 3.2 Similar expression profiles of *FT* orthologs in different photoperiods

Due to the limited information available for SDPs, we summarized *FT* orthologs from two SDPs in this review. Therefore, we cannot generalize the expression profiles of *FT* orthologs of SDPs. However, we observed that all *FT* orthologs of SDPs examined in this review are expressed only in the morning, even under LD conditions, which differs from the expression profiles of *FT* orthologs in LDPs (**Table 1**, **Figure 2**). This observation can be further categorized into two cases; 1) different *FT* orthologs are expressed with similar profiles under different photoperiod, as seen in the case of soybean. 2) the same *FT* orthologs are expressed with the same profiles under different photoperiods, although the expression levels differ, as seen in the case of rice. In soybean, the peak expression time of floral activators (*GmFT2a* and *GmFT5a*) under SD conditions coincides with that of floral repressors (*GmFT1a* and *GmFT4*) under LD conditions. Rice *FT* orthologs, *Hd3a* and *RFT1*, peak only once in a day in the morning, both in SD and in LD. According to the prevailing model, photoperiod-dependent on-off expression could be explained by the functionality of major regulators, E1 in soybean and Hd1 and Ehd1 in rice. However, the time-of-day-dependent expression profiles of *FT* orthologs remain elusive. Interestingly, both plant species possess dual-function regulators: E1 in soybean and Hd1 in rice. In rice, the activity of Hd1 on *Hd3a* expression in LD is mediated by its interaction with DAYS TO HEADING 8 (DTH8) (Du et al., 2017). In SD, the Hd1-DTH8 complex is likely absent due to low expression of *DTH8*, resulting in the activation of *Hd3a* expression. Considering that E1 has been shown to possess transcriptional repression ability (Zhai et al., 2022), the activity of E1 is likely regulated by its binding partner(s). Additionally, both plant species have their unique regulatory factors: the legume-specific E1 and the rice-specific Ehd1. These pieces of evidence suggest that regulatory factors involved in the photoperiod-dependent expression of *FT* orthologs in SDPs remain undiscovered. Investigating whether SDPs have conserved regulatory mechanisms that confer the unique expression profiles of *FT* orthologs in SDPs would help expand our knowledge about the photoperiodic flowering regulation.

### 3.3 Multilayered regulatory mechanisms of *FT* transcript levels

Transcript levels are determined not only by *de novo* synthesis of mRNA but also by mRNA stability. However, quantitative RT-PCR, widely used for quantification of transcript levels, measures endpoint transcript levels, thus having a limitation in distinguishing between mRNA synthesis and its stability regulation. In *Arabidopsis*, *FT* mRNA stability is non-cell autonomously regulated by WEREWOLF (WER), an R2R3 MYB transcription factor (Seo et al., 2011). WER likely regulates the abundance of *FT* mRNA by stabilizing the transcripts rather than by increasing transcription. The *wer* mutant plants exhibited lower *FT* transcript levels compared to wild type, consequently displaying a late-flowering phenotype in a photoperiod-dependent manner (Seo et al., 2011). However, *WER* is expressed in both LD and SD conditions, and both *WER* mRNA and WER protein are mainly observed in the epidermis. These findings indicate that other factors are likely involved in the WER-mediated stabilization of *FT* mRNA. Therefore, further investigation is necessary to determine whether the photoperiod-dependent expression profiles of *FT* and its orthologs are shaped by *de novo* transcription and/or mRNA stability.

In addition to *Arabidopsis FT*, *FT* orthologs in *Brachypodium distachyon* and *Cocos nucifera* have been shown to be regulated at the post-transcriptional level (Wu et al., 2013; Qin et al., 2017; Xia et al., 2020). mRNAs of two *FT* orthologs in *Brachypodium*, *BdFT1* and *BdFT2*, are targeted by miR5200 for mRNA cleavage (Wu et al., 2013). miR5200 expression is higher in SD conditions than in LD conditions, thus it inhibits the accumulation of *BdFT1* and *BdFT2* specifically in SD. Additionally, *BdFT2* generates two different splicing isoforms, named *BdFT2α* and *BdFT2β*. *BdFT2β* lost its ability to bind to FD but retained the ability to form a heterodimer with *BdFT2α* or *BdFT1*, thereby resulting in attenuated formation of flowering inductive complexes (Qin et al., 2017). The mode of action derived by alternative splicing of *BdFT2* is affected by an endogenous cue, as shown by gradual decrease in the *BdFT2β*/*BdFT2α* ratio as development progresses (Qin et al., 2017). Alternative splicing of *FT* orthologs has also been observed in wheat, barley and coconut (Qin et al., 2017; Xia et al., 2020). However, it remains unclear whether the alternative splicing of *FT* orthologs is photoperiod dependently regulated. Nevertheless, the accumulating evidence suggests that the mechanisms underlying the post-transcriptional regulation of *FT* transcripts may be conserved and regulated not only by endogenous cues but also environmental cues, such as photoperiod.

## 4 Conclusions

Since the identification of FT as a long-sought florigen in *Arabidopsis*, numerous studies have contributed to establishing the molecular mechanisms underlying how *FT* expression is elaborately regulated to determine flowering time. However, expression profiles of *Arabidopsis FT* appear to be differentially determined depending on light quality (Song et al., 2018; Lee et al., 2023). The current model hardly explains the bimodal *FT* expression profile in nature, particularly the morning *FT* expression. In this review, we aimed to gather and explore expression profiles of *FT* orthologs to gain insight into underlying regulatory mechanisms. Despite limitations in publicly available information for diurnal expression of *FT* orthologs in diverse plants, expression profiles of *FT* orthologs combined with their functional activity in flowering time regulation suggest that plants might share conserved regulatory mechanisms for the expression of *FT* orthologs. Firstly, *FT* orthologs tend to highly express in facultative photoperiodic conditions, as expected; high expression of *FT* orthologs of LDPs in LD conditions, while high expression of *FT* orthologs of SDPs in SD condition. Secondly, plants classified in the same type of photoperiodic flowering responses likely show similar expression profiles of *FT* orthologs; *FT* orthologs of LDPs peak either once in the late afternoon or twice in the morning and the evening in LD conditions, while *FT* orthologs of SDPs peak in the morning in SD conditions. Thus, not only *FT* orthologs per se but also regulatory mechanisms for the expression of *FT* orthologs may be conserved among plant species showing the same photoperiodic responses in flowering. However, it should be noted that, although *FT* orthologs in many plants are believed to function as florigens to promote flowering, several *FT* orthologs have shown to function as a repressor, which diversifies expression profiles of *FT* orthologs. The *FT*-like floral repressors examined in this review could be classified into two groups, based on their expression profiles. One consists of *FT*-like floral repressors that antagonistically express against the *FT*-like floral activators. These opposite expression patterns match with their role in flowering regulation to induce flowering only under favorable conditions. Another group contains *FT*-like floral repressors whose expression coincides with expression of *FT*-like floral activators in the same photoperiod and/or at the time of day. It remains unclear whether these FT-like floral repressors counteract the FT-like floral activators for flowering in the same conditions. Given the functional diversification of FT orthologs beyond floral regulation (Wang et al., 2022), it is possible that co-expression of *FT*-like floral activators and *FT*-like floral repressors may differentially contribute to processes other than flowering. Further exploration of redundancy among *FT* orthologs may give us clues to the significance of co-expression of *FT* orthologs.

Advanced molecular biology techniques, coupled with bioinformatics, has facilitated in-depth exploration of flowering across diverse plant species. Moreover, sophisticated analyses using *Arabidopsis* has opened a new chapter for regulatory mechanisms of *FT* expression in nature. The *FT* orthologs across plant species could be classified according to the similarity of expression profiles. Thus, comparative analyses within and between plant species might offer insights into molecular mechanisms. The effect of phyA or FR light on expression profiles of *FT* orthologs in plants beyond *Arabidopsis* has received less attention. Most plants examined in this review respond to the supplementary FR light to promote flowering and phyA functions as a positive regulator, although soybean has shown the opposite effect of phyA and FR light on flowering. Investigation into the interplay between FR light (and phyA) and the expression of individual *FT* orthologs may be helpful to understand flowering in diverse plant species in their natural environments.

## Author contributions

NL: Funding acquisition, Writing – original draft, Writing – review & editing. JS: Writing – review & editing, Funding acquisition. M-KK: Writing – review & editing. MK: Funding acquisition, Writing – original draft, Writing – review & editing.
